# The Assessment of Oral Microflora Exposed to 3% Ethanolic Extract of Brazilian Green Propolis Preparation Used for Hygiene Maintenance following Minor Oral Surgeries

**DOI:** 10.1155/2015/869575

**Published:** 2015-08-26

**Authors:** Tadeusz Morawiec, Anna Mertas, Robert D. Wojtyczka, Iwona Niedzielska, Arkadiusz Dziedzic, Anna Bubiłek-Bogacz, Jakub Sender, Jacek Wróbel, Marta Tanasiewicz, Piotr Wesołowski, Wojciech Król

**Affiliations:** ^1^Department of Oral and Maxillo-Facial Surgery, School of Medicine with the Division of Dentistry in Zabrze, Medical University of Silesia in Katowice, Plac Akademicki 17, 41-902 Bytom, Poland; ^2^Department of Microbiology and Immunology, School of Medicine with the Division of Dentistry in Zabrze, Medical University of Silesia in Katowice, Jordana 19, 41-808 Zabrze, Poland; ^3^Department and Institute of Microbiology and Virology, School of Pharmacy and Laboratory Medicine, Medical University of Silesia in Katowice, Jagiellońska 4, 41-200 Sosnowiec, Poland; ^4^Department of Conservative Dentistry with Endodontics, School of Medicine with the Division of Dentistry in Zabrze, Medical University of Silesia in Katowice, Plac Akademicki 17, 41-902 Bytom, Poland; ^5^Department of Oral Surgery, Medical University in Warsaw, Nowogrodzka 59, 02-006 Warsaw, Poland

## Abstract

The aim of this study was to investigate the influence of a topically administered hygienic preparation containing a 3% ethanolic extract of Brazilian green propolis (EEP-B) on oral microflora spectrum changes in a group of patients who underwent common oral surgery procedures. Two gel samples were compared: the tested gel containing an active ingredient, that is, a 3% EEP-B (gel GA), and a placebo as the negative control (gel GC). The collection of microbiological material included 14 patients requiring surgical extraction of wisdom molars and short endosseous implant installation. Clinical examinations were carried out as follow-up, that is, baseline and after 5-6 weeks' time. During the first and subsequent assessment, swabs were taken from the mucosal surface. The number of microorganism species was found to have increased following the application of GC gel over the period of 5-6 weeks. This mainly affected Gram-positive rods and bacilli as well as Gram-negative rods. Application of the GA gel enriched with 3% EEP-B caused a profound reduction in the amount of *Neisseria* spp. and *Bifidobacterium* spp. strains. Elimination of seven species of microorganisms was observed: *Streptococcus acidominimus, Streptococcus oralis, Staphylococcus epidermidis, Veillonella parvula, Bifidobacterium breve, Bifidobacterium longum, and Lactobacillus acidophilus*.

## 1. Introduction

Optimal oral hygiene is one of the conditions influencing uncomplicated restitution of operated areas, with a strong impact on postoperative healing of alveolar structures in patients who have undertaken minor surgery procedures within the oral cavity [1, 2]. A strict oral hygiene regime must be maintained for at least 7 days after the procedure, to make the patient feel comfortable and safe [3, 4]. The preparations applied topically within the oral cavity, containing organic substances and antiseptic agents, including gels or mouthwashes, are widely known for their bactericidal and anti-inflammatory properties [5–9].

Invasive dental procedures related to common oral surgery (e.g., third molar extractions) favor bacterial dissemination, causing postoperative inflammatory reactions [10–12]. They may depend on the severity of the bacterial load, the duration of microbial exposure, the type of bacterial predominance (aerobic, anaerobic, or mixed), and the patient's predisposition, including underlying diseases and individual susceptibility to infection [13, 14]. These factors play a significant role in the onset of possible postoperative complications, which may be triggered by commensal pathogens and their toxins in specific circumstances [15]. The colonization of microflora on various oral surfaces may result most notably in the incidence of postoperative opportunistic infections, where the surrounding soft tissues become inflamed as a result of exposure to the bacteria and yeasts present in saliva [16, 17]. Opportunistic infections arise due to an imbalance in the conditions of the oral cavity, such as immunological suppression and general health conditions affecting the oral environment [18], or due to compromised oral structures, which expose the vulnerable oral mucosa to microorganisms [19, 20]. Patients who do not maintain proper oral hygiene are more susceptible to imbalances in microflora and opportunistic infections. Bacteria in the oral microflora may become the etiological factor in other focal microbial infections, for example, infective endocarditis [20], which can sometimes develop into life-threatening emergencies if not treated promptly and effectively. The antibiotic susceptibility pattern of some oral pathogens may make the selection of an effective chemotherapeutic regimen difficult [21]. Moreover, strains isolated from oral infections are frequently resistant to standard synthetic antibacterial agents. Since many reports have shown that antibiotics are often ineffective in the eradication of oral biofilm, further studies regarding biological agents, including natural organic substances, may support the need for alternative antibacterial protocols to be applied for the treatment of refractory infections caused by oral microorganisms [9, 22].

Propolis, a natural compound, is a wax/resin mixture used by bees to seal up holes or slits in their beehives. It is probably collected by bees from tree buds or other green plants or it may be a pollen product secreted by bees as indigestible material [23]. Complex propolis composition varies according to its origin [24, 25] and usually contains resins (40%), waxes (23–30%), polyphenols (14–16%), polysaccharides (2.5%), volatile matters (>10%), and mechanical additives [23, 26, 27]. A number of propolis preparations, showing biological activity, have been obtained through organic solvent extraction. Among these solvents, ethanol is the most commonly used, and the ethanolic extract of propolis (EEP) has wide practical applications [28, 29].

This study aimed to determine the antimicrobial effect of the hygienic preparation (gel) containing 3% ethanolic extract of Brazilian green propolis (EEP-B) on the oral microflora spectrum changes, in the context of postoperative prevention of surgical complications, in a group of outpatients who underwent common oral surgery procedures, including extraction of third molars and a single installation of short endosseous implants.

## 2. Material and Methods

### 2.1. Propolis

Raw propolis was collected from the beekeeping section of the Seiri Alimentos Naturais, Brazil. Propolis samples were obtained from colonies of Africanized honeybees (*Apis mellifera*) in Minas Gerais State, southeast Brazil. Green propolis collected in the southern region of Brazil belongs to Group 12 (propolis G12), as twelve distinct groups of Brazilian propolis have been classified according to their botanical origin and biological properties: five from the south, six from the northeast, and one from the southeast named propolis “green” [26, 30]. However, only three types of Brazilian propolis had their botanical origin and chemical constituents identified [31]. One of these confirmed propolis types is the studied green propolis from Minas Gerais State in southeast Brazil, which is derived mainly from alecrim plants* Baccharis dracunculifolia* (Asteraceae).* Baccharis*, which contains more than 500 species, appears to be a cosmopolitan genus distributed in South, Central, and North America. Large populations of* Baccharis* species are present in the field vegetation in Brazil [32].

The unprocessed Brazilian green propolis was sent to the Nihon Natural Therapy Co. Ltd. (Tokyo, Japan) for preparation of the propolis extract. Propolis was extracted in 95% V/V ethyl alcohol, in a hermetically sealed glass vessel, for 4 days, at 37°C, under occasional shaking. The ethanolic extract of Brazilian green propolis (EEP-B) was then filtered and evaporated under reduced pressure at 60°C. Chemical evidence based on previously described [33, 34] high-performance liquid chromatography (HPLC-DAD) analysis suggested that the main flavonoid compounds presented in EEP-B were kaempferol and quercetin, as well as other ingredients: cinnamic acid derivatives such as *p*-coumaric acid and artepillin C. The gel with 3% EEP-B (GA gel) and without EEP-B (GC gel—placebo), used in this study, was prepared by Nippon Zettoc Co., Ltd. (Tokyo, Japan).

### 2.2. Patients

This clinical study was carried out to investigate the influence of a propolis-based gel on the postoperative process of oral soft tissue repair and oral microbiota spectrum changes. This research was conducted between December 1, 2012, and March 1, 2013, in the Oral Surgery Department at the Academic Centre of Dentistry and Specialist Medicine in Bytom and in the Specialist Dental Clinic in Katowice, which provide specialist emergency and planned dental care for patients requiring minor oral surgery procedures, including surgical wisdom tooth extraction (partially erupted or fully impacted) and short endosseous implant installation. The study included 14 outpatients (seven men and seven women) aged 18–48 years. All of them came from cities of the Silesian macroregion cities. Subgroup GA (gel preparation with 3% EEP-B) included seven patients (three men and four women), while subgroup GC (gel preparation without propolis as a negative control) included the same number of seven patients (four men and three women).

Patients qualification for the study was based on medical and dental history, interviews, and a review of the clinical records. Patients selected were free of systemic illnesses, did not present with acute infection at the surgical site, and did not take antibiotics for at least two weeks before surgery. All patients were informed on the purpose of the study and agreed to participate in it. The criteria for exclusion from the investigation were lack of patient's valid consent, medically compromised patients, inability to comply with the follow-up visit requirements, patients receiving concurrent antibiotic treatment for any other purpose, individuals with confirmed adverse reactions to bee products, nursing or pregnant women, and recent postoperative oral surgery cases. The research programme was approved by the Bioethics Committee of the Silesian Chamber of Medicine (Resolution number 6/2000, dated 01.03.2000).

### 2.3. Clinical Protocol

Surgical procedures were performed by three operators, specialists in oral surgery or registrars in oral surgery. In all cases, the inferior alveolar, lingual, and buccal nerves were anesthetized with two anesthetic cartridges of 2% lidocaine with 1 : 50000 epinephrine (Xylestesin, 3MESPE, Germany) or 4% articaine with 1 : 200000 epinephrine (Septanest, Septodont, France). As in the vast majority of cases of surgical wisdom tooth removal, standard trajectory incisions were made along the retromolar area to the second molar and another incision was made as a vertical releasing incision on the mesial side of the lower second molar. Bone removal was performed using a surgical bur as needed. After extraction of the tooth, the socket was cleaned and any solid remnants were removed.

Fourteen patients were randomly assigned to two groups of seven subjects each, which received an unlabeled GA gel or a negative control GC gel. Each patient was given the gel in an unlabeled packet. Preparations with propolis or without propolis were assigned at random. The investigator did not know the contents of the packets either. Oral hygiene instructions were given in an attempt to improve the subjects' oral hygiene before entry into the study. All patients received professional advice regarding oral hygiene and were instructed to brush their teeth at least two times a day with the gel for at least two minutes and to refrain from all other oral hygiene measures until the next examination.

At the first visit (on the day of the surgery), the history was taken and a clinical examination was performed, the latter including an assessment of the dentition. A sample was taken from the mouth floor mucosa for microbiological testing. The patient was instructed as regards oral hygiene. During the reassessment appointment (day 7 after surgery) the sutures were removed. At the first visit, the history was taken, concerning eating habits, consumption of tea, coffee, and alcoholic beverages, regular appointments at the dentist, and frequency of cleaning the teeth. Also, some questions were asked about education and financial status. A standard swab for microbiological examination was taken from the mucosal surface of the region where surgical extraction of the impacted tooth or implant installation was to be performed. Following the surgery, each patient received a packet of a gel with no name on it and was instructed to use it twice a day. In addition, brushing the teeth by the Fones method was recommended. The patients did not use any other means or methods for cleaning their teeth throughout the study. Postoperative care was given.

The second visit (5-6 weeks after surgery) consisted of clinical examinations and swabbing for microbiological testing. The history was taken with special attention paid to how many times per day the teeth were cleaned to maintain proper oral hygiene. Clinical examination included an assessment of oral hygiene, the periodontium, and the mucosa. Samples of biological material were taken from the postoperative region for microbiological testing.

### 2.4. Microbiological Investigation

Microbiological tests were performed by the Department of Microbiology and Immunology in Zabrze of the Medical University of Silesia in Katowice. The samples were inoculated on suitable culture media (Columbia agar, Schaedler K3 agar, and Sabouraud agar) from bioMerieux (Marcy l'Etoile, France). Aerobic bacteria were propagated on Columbia agar solid medium with 5% sheep blood at 37°C. Anaerobic bacteria were propagated on Schaedler K3 solid medium with 5% sheep blood at 37°C under anaerobic conditions using a GENbag Anaer (bioMerieux, Marcy l'Etoile, France).* Candida* fungi were propagated on selective Sabouraud agar solid medium at 35°C under aerobic conditions. Upon isolation and further culture of each microorganism, their species were identified by the following tests: Api 20 E, Api 20 NE, and Api* Candida* (bioMerieux, Marcy l'Etoile, France) and ENTEROtest 24 N, NEFERMtest 24 N, STREPTOtest 24, STAPHYtest 24, and ANAEROtest 23 (Erba-Lachema, Brno, Czech Republic).

The data from individual patients were treated as confidential and were not identifiable in any documentation that emerged in relation to the examination. The study represented a separate part of the main research project at the Medical University of Silesia supported by the Grant KNW-2-102/10 and was performed following the guidelines of the Declaration of Helsinki.

### 2.5. The Statistical Analysis

The statistical differences between groups were determined by analysis of variance followed by the unpaired Student *t*-test and the Mann-Whitney *U* test, depending on how well the results correlated with a normal distribution. Differences between the mean values were considered to be statistically significant at *p* < 0.05. The STATISTICA version 10 software (StatSoft, Cracow, Poland) was used to perform the statistical analysis.

## 3. Results

Fourteen patients successfully completed the study according to the research protocol. There were seven male (50%) and seven (50%) female patients, with a mean age of 39.7 years. All patients presented with a single region procedure. The mandibular retromolar triangle was the most frequent location for a single-space odontogenic problem (*pericoronitis*)—71.5%—followed by the anterior mandibular alveolar region (short dental implants installation)—28.5%. The dental implants were designed as a fixed, endosseous support for prosthodontic overdentures.

Microbiological testing of the samples harvested from the surgical area of the seven patients using the GA gel with 3% EEP-B for 5-6 weeks detected a smaller number of microorganism isolates as compared to the first microbiological test performed prior to using the GA gel. Test I revealed 29 microorganism isolates representing 14 species, whereas test II (after 5-6 weeks) revealed 23 microorganism isolates representing 16 species (Table 1).

The following observations were made:

- elimination of six bacterial species:* Streptococcus acidominimus*,* Streptococcus oralis*,* Staphylococcus epidermidis*,* Veillonella parvula*, and* Bifidobacterium breve*, all of them appearing in the mouth microflora, and* Lactobacillus acidophilus*, being cariogenic, and their removal certainly made a positive effect;
- enrichment of the mouth microflora by eight new microorganisms:* Streptococcus sanguinis*,* Staphylococcus aureus*, and* Bifidobacterium dentium*, appearing in the physiological mouth microflora;* Aeromonas caviae*, appearing in water and damp environments, originating in contaminated water or food, likely to cause infection of wounds and connective tissue;* Actinomyces israelii*, appearing in the mouth microflora, an etiological factor of actinomycosis;* Campylobacter gracilis*, its chief reservoir being animals, but pathogenic for humans, that is, gastroenteritis, systemic infections, septic thrombophlebitis, arthritis, and cerebrospinal meningitis;* Enterobacter kobei*, widely common in the environment, causing hospital infections, particularly wound infections;* Klebsiella pneumoniae*, appearing often in the gastrointestinal tract microflora as an opportunistic pathogen (pneumonia, hospital infections).

Table 2 presents the percentage of the main isolated species in the propolis GA group and the control GC group. The application of GA gel enriched with propolis extract caused a profound reduction in the number of Gram-positive anaerobes.

As far as the other isolated microorganisms are concerned (*Bifidobacterium longum*,* Sarcina *sp., and* Candida albicans*), an identical number of isolates were detected by both microbiological tests. The effect of propolis on* Candida albicans* was distinctive and nonsignificant.* C. albicans* was isolated by test I in five patients and by test II in four of them and in one new patient, which means that this microorganism was only eliminated in one patient from the oral cavity microflora. Analysis of the influence of propolis gel on the mouth microflora showed beneficial changes in quantity. Test II revealed fewer microorganism isolates (by six) than test I, and the quality of the composition improved through eliminating potential bacterial pathogens while maintaining the proper composition of the physiological flora.

On the other hand, no such beneficial changes were observed in the group of seven patients who used the GC gel without propolis for oral hygiene. Quality changes were fairly similar to those observed in patients who used the propolis gel. After 5-6 weeks of using the propolis-free preparation, an increased number of microorganism isolates were detected. Test I on the sample harvested from surgical areas revealed 26 isolates of 13 species, and test II revealed 29 isolates representing 15 different species. The second microbiological test revealed the following changes:

- elimination of six microorganism species:* Streptococcus oralis*,* Streptococcus vestibularis*, and* Staphylococcus epidermidis MSCNS*, appearing in the mouth physiological microflora;* Actinomyces viscosus*, responsible for parodontopathies and the development of dental caries;* Burkholderia cepacia*, responsible for opportunistic hospital infections, including respiratory tract infections;* Klebsiella oxytoca*, likely to appear in gastrointestinal tract microflora, as an opportunistic pathogen (pneumonia, hospital infections);
- enrichment of the mouth microflora by eight new species:* Ruminococcus productus*,* Sarcina *sp.,* Bifidobacterium adolescentis*,* Bifidobacterium dentium*,* Capnocytophaga ochracea*, and* Enterobacter amnigenus*, widely common in the environment, causing nosocomial infections, chiefly wound infections;* Klebsiella pneumoniae*, likely to appear in the gastrointestinal tract microflora, as an opportunistic pathogen (pneumonia, hospital infections);* Prevotella disiens*, appearing in the mouth microflora, likely to cause gingivitis, pharyngitis, lower airway inflammation, and head or neck abscesses.

Furthermore, test II performed on patients using the gel GC without propolis showed a smaller number of* Staphylococcus aureus (MSSA)* and* Neisseria *sp. isolates. In the case of the other isolated microorganisms (*Streptococcus mitis*,* Streptococcus sanguinis*,* Streptococcus salivarius*,* Veillonella parvula*, and* Candida albicans*), an identical number of isolates were detected in both microbiological tests. No beneficial effects of propolis leading to the elimination of the fungus* Candida albicans* from the mouth microflora were shown. Test I allowed the isolation of* C. albicans *in three patients and test II revealed* C. albicans* in the same three patients.

Microbiological tests performed on 28 samples collected from the oral cavity revealed 107 isolated microorganisms. They represented 29 species, which were later divided into the following groups: Gram-positive cocci, which contained* Streptococcus mitis*,* Streptococcus oralis*,* Streptococcus sanguinis*,* Streptococcus salivarius*,* Streptococcus vestibularis*,* Streptococcus acidominimus*,* Staphylococcus aureus*,* Staphylococcus epidermidis*,* Ruminococcus productus*, and* Sarcina* sp.; Gram-negative cocci, such as* Neisseria *spp.,* Veillonella parvula*; Gram-negative rods, such as* Klebsiella pneumoniae*,* Klebsiella oxytoca*,* Enterobacter amnigenus*,* Enterobacter kobei*,* Burkholderia cepacia*,* Capnocytophaga ochracea*,* Campylobacter gracilis*, and* Prevotella disiens*; and Gram-positive rods and bacilli, such as* Bifidobacterium adolescentis*,* Bifidobacterium dentium*,* Bifidobacterium breve*,* Bifidobacterium longum*,* Lactobacillus acidophilus*,* Aeromonas caviae*,* Actinomyces viscosus*, and* Actinomyces israelii*, as well as the fungus* Candida albicans*.

**Figure 1:**
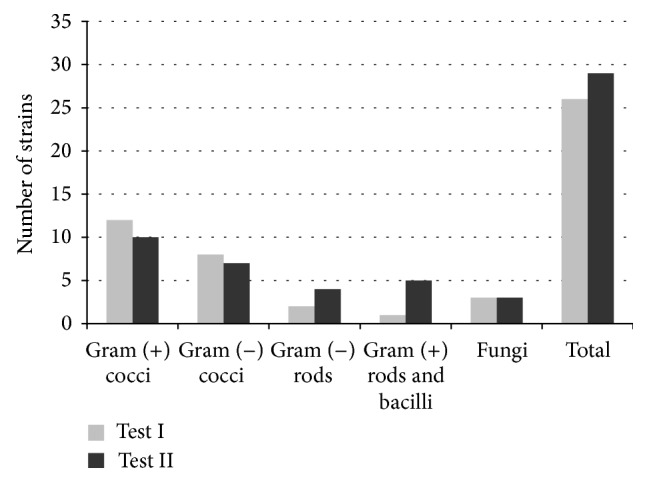
Graphical representation of microorganism strain changes for the GC preparation (without propolis).

In the control group (patients using the gel GC without propolis), the number of species of microorganisms was found to have increased, in comparison with the swabs collected before the preparation was applied. The increase mainly affected Gram-positive rods and bacilli, as well as Gram-negative rods. The amount of yeast-like fungi of the* Candida albicans* type remained stable (Table 3, Figure 1).

**Figure 2:**
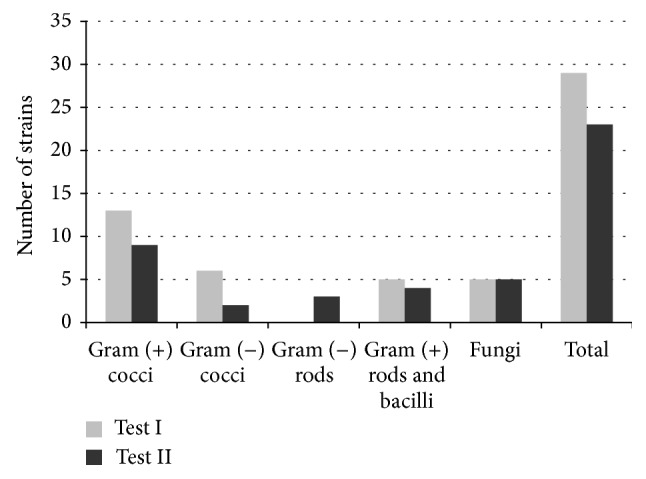
Graphical representation of microorganism strain changes for the GA preparation (with 3% EEP-B).

By analyzing the results of quantitative studies in patients applying the gel with the addition of propolis for six weeks, one can note that the number of Gram-positive and Gram-negative micrococci diminished substantially. In the second test, Gram-negative rods of Enterobacteriaceae appeared. The amount of* Candida albicans* fungi remained stable (Table 3, Figure 2). After analyzing the qualitative studies of mouth cavity swabs in patients applying the GC and GA gels, it was found that, in the case of applying the gel without the addition of propolis, the analyzed groups of microorganisms contained mainly the following strains:* Bifidobacterium adolescentis*,* Bifidobacterium dentium *as well as single strains of* Enterobacter amnigenus*,* Klebsiella pneumoniae*,* Prevotella disiens*,* Capnocytophaga ochracea*,* Ruminococcus productus*, and* Sarcina *sp., which had not been present when treatment commenced. In these studies carried, six species were eliminated, of such strains, including* Streptococcus oralis*,* Streptococcus vestibularis*,* Staphylococcus epidermidis*,* Actinomyces viscosus*,* Burkholderia cepacia*, and* Klebsiella oxytoca*.

Assessing the species changes in the bacterial flora in the course of application of the gel with propolis, the most profound reduction in the amount of microorganisms was achieved in the case of the strains* Neisseria* spp. and* Bifidobacterium* spp. After six weeks of applying the gel with propolis to the patients, the elimination of seven species of microorganisms was observed, namely,* Streptococcus acidominimus*,* Streptococcus oralis*,* Staphylococcus epidermidis*,* Veillonella parvula*,* Bifidobacterium breve*,* Bifidobacterium longum*, and* Lactobacillus acidophilus*. As in the case of applying the GC gel, single strains appeared, mainly belonging to the Gram-negative rods, such as* Klebsiella pneumoniae*,* Enterobacter kobei*,* Campylobacter gracilis*, or other species, such as* Streptococcus sanguinis*,* Staphylococcus aureus*,* Aeromonas caviae*,* Bifidobacterium dentium*, and* Actinomyces israelii*. In both cases (with the application of the GC or GA gels), no changes were observed as regards the number of yeast-like fungi of the* Candida albicans* type (Table 1).

## 4. Discussion

In clinical applications, EEP has been shown to have regenerative effects, and this observation has been confirmed by a number of experiments. It has been demonstrated that local application of a substance containing propolis encourages the healing of the wounds through reducing inflammation and relieving pain after oral surgery. Magro-Filho and de Carvalho observed that topical application of a propolis hydroalcoholic solution accelerated epithelial repair after tooth extraction but had no effect on socket wound healing [35]. Good therapeutic effects of EEP have also been observed in oral medicine, in cases of dry sockets and parodontopathies [36–38]. Al-Sultan et al. concluded that an aqueous extract of propolis as a topical agent following lower third molar extraction had a slight reducing effect on the severity of postoperative complications [39]. It was observed that Brazilian propolis mouthrinse was effective in suppressing cariogenic infections as well as reducing gingival inflammation [40, 41]. There is a granted patent in Brazil about products elaborated with Brazilian green propolis for use in dentistry [42].

EEP has bactericidal [43, 44], fungicidal [45, 46], anti-inflammatory [47, 48], and antioxidative properties, as well as the ability to scavenge free radicals [24, 49]. Nowadays, propolis extract is used as an addition to oral care preparations (toothpastes, mouthwashes, and prophylactic gels) to enhance their antibacterial, disinfecting, and anti-inflammatory effects. Propolis has been found to have an anti-inflammatory effect through the inhibition of cyclooxygenase (COX-2) and consequent inhibition of prostaglandin biosynthesis (PGE_2_) and the ability to scavenge free radicals produced by neutrophils and macrophages, inhibit inducible nitric oxide synthase (iNOS), reduce the concentration of inflammatory cytokines (IL-1*β*, IL-2, IL-6, IL-10, and TGF-*β*), and possess immunosuppressive activity [50–52]. Apart from the reduction of acute and chronic inflammatory conditions, propolis accelerates the formation of granulation tissue and epithelium [53].

Most microorganisms involved in postoperative infections of the head and neck are of odontogenic origin [54, 55]. Bacteria that were isolated consisted of both aerobic and anaerobic organisms. The results of the present clinical study show the effectiveness of a topical hygienic preparation containing a 3% ethanol extract of Brazilian green propolis (EEP-B) against facultative anaerobic oral microorganisms. However, infections due to anaerobic and Gram-negative organisms have increased over the last decade in comparison with past reports in the dental literature [13]. This may be related to improvements in isolating and culturing methods of anaerobic organisms from the oral cavity. Our study showed a predominance in aerobic (strict and facultative) over anaerobic species isolated. Gram-positive cocci were the predominant bacteria cultured from our specimens and Gram-negative rods were the second most common bacteria isolated. This is consistent with the results of other studies [56–58].

Recent studies provide new evidence-based support for the antimicrobial activity of Brazilian green propolis extract against a range of oral bacteria [34, 36, 43, 44, 59, 60]. Koru et al. investigated the antibacterial efficiency of propolis against certain anaerobic oral pathogens and found it to be very effective against* Peptostreptococcus anaerobius*,* Lactobacillus acidophilus*,* Actinomyces naeslundii*,* Prevotella oralis*,* Prevotella melaninogenica*,* Porphyromonas gingivalis*,* Fusobacterium nucleatum*, and* Veillonella parvula* [61]. They concluded that the antibacterial property of propolis is due to the presence of flavonoids and aromatic compounds such as cinnamic acid.

According to the broad literature, the biologically active molecules in green propolis are phenolic acids and flavonoids, which act as scavengers of free radicals and inhibitors of nitric oxide and inflammatory cytokines production by macrophages and neutrophils [49–52]. Kaempferide and its derivatives and cinnamic acid derivatives, *p*-coumaric acid and artepillin C, were the major constituents identified in a tested sample of Brazilian green propolis extract [30, 32, 33]. Hayashi et al. observed significant antioxidant effects of kaempferide and artepillin C, compounds isolated from Brazilian propolis [62]. The results of other studies suggest a contribution of Brazilian green propolis in the modulation of chemokine-mediated inflammation, which also exhibits antioxidant properties by scavenging reactive oxygen species and inhibiting chemiluminescence reactions [48, 63]. These biological effects of propolis compounds have a significant, direct impact on the viability of the oral microflora, including the elimination of pathological microorganisms. It can be assumed that the combined anti-inflammatory and antibacterial effects of propolis play an important role in the prevention of postoperative complications in dental patients after extensive, alveolar procedures.

## 5. Conclusion

Hygienic preparations enriched with propolis extract might be used as a natural alternative or additive to chemical means during the postoperative period associated with oral surgery procedures. Topical, antibacterial prophylaxis for surgical dental procedures is recommended when the highest risk of occurrence of postoperative complications is expected in patients who have undergone invasive dental procedures. Maintaining optimal oral hygiene, supported by antiseptic topical measures (mouthwash, toothpaste, and gel), is fundamental in the prevention of alveolar wound infections and in the majority of clinical cases seems to be more important than antibiotic pharmacotherapy.

## Figures and Tables

**Table 1:** Changes in oral microflora of patients using gel GC without propolis and those using gel GA with the addition of 3% EEP-B.

Isolated microorganisms	Number of isolated strains			
	GC gel (*n* = 7)		GA gel (*n* = 7)	
	Test I	Test II	Test I	Test II
Gram (+)				
*Streptococcus mitis*	5	5	4	3
*Streptococcus oralis*	1	0	1	0
*Streptococcus sanguinis*	1	1	0	1
*Streptococcus salivarius*	1	1	3	2
*Streptococcus vestibularis*	1	0	2	1
*Streptococcus acidominimus*	0	0	1	0
*Staphylococcus aureus MSSA*	2	1	0	1
*Staphylococcus epidermidis MSCNS*	1	0	1	0
*Ruminococcus productus*	0	1	0	0
*Sarcina *sp.	0	1	1	1
Gram (−)				
*Neisseria *spp.	7	6	5	2
*Veillonella parvula*	1	1	1	0
*Aeromonas caviae*	0	0	0	1
*Bifidobacterium adolescentis*	0	3	2	1
*Bifidobacterium breve*	0	0	1	0
*Bifidobacterium dentium*	0	2	0	1
*Bifidobacterium longum*	0	0	1	0
*Lactobacillus acidophilus*	0	0	1	0
*Actinomyces viscosus*	1	0	0	0
*Actinomyces israelii*	0	0	0	1
*Burkholderia cepacia*	1	0	0	0
*Capnocytophaga ochracea*	0	1	0	0
*Campylobacter gracilis*	0	0	0	1
*Enterobacter amnigenus*	0	1	0	0
*Enterobacter kobei*	0	0	0	1
*Klebsiella oxytoca*	1	0	0	0
*Klebsiella pneumoniae*	0	1	0	1
*Prevotella disiens*	0	1	0	0
Fungi				
*Candida albicans*	3	3	5	5
Total number of strains	**26**	**29**	**29**	**23**

Test I—sample collected before GC or GA gel application (baseline).

Test II—sample collected 5-6 weeks following GC or GA gel application (final assessment).

**Table 2:** Percentage of isolated microorganisms strains in propolis group, GA, and control group, GC (baseline—test I, final assessment—test II).

Isolated microorganisms	GC gel (*n* = 7)		GA gel (*n* = 7)	
	Test I [%]	Test II [%]	Test I [%]	Test II [%]
Gram (+) facultative anaerobes				
*Streptococcus mitis*	19.2	17.25	13.79	13.04
*Streptococcus oralis*	3.85	0.00	3.44	0.00
*Streptococcus sanguinis*	3.85	3.45	0.00	4.34
*Streptococcus salivarius*	3.85	3.45	10.34	8.69
*Streptococcus vestibularis*	3.85	0.00	6.89	4.34
*Streptococcus acidominimus*	0.00	0.00	3.44	0.00
*Staphylococcus aureus MSSA*	7.70	3.45	0.00	4.34
*Staphylococcus epidermidis MSCNS*	3.85	0.00	3.44	0.00
*Actinomyces viscosus*	3.85	0.00	0.00	0.00
*Actinomyces israelii*	0.00	0.00	0.00	3.34
*Lactobacillus acidophilus*	0.00	0.00	3.44	0.00
	**50.00**	27.6^**∗**^	**44.8**	39.1^**∗**^
Gram (+) anaerobes				
*Ruminococcus productus*	0.00	3.45	0.00	0.00
*Sarcina *sp.	0.00	3.45	3.44	4.34
*Bifidobacterium adolescentis*	0.00	10.35	6.89	4.34
*Bifidobacterium breve*	0.00	0.00	3.44	0.00
*Bifidobacterium dentium*	0.00	6.9	0.00	4.34
*Bifidobacterium longum*	0.00	0.00	3.44	0.00
	**0.00**	24.1^**∗**^	**17.25**	**13.0**
Gram (−) facultative anaerobes				
*Neisseria *spp.	27.0	20.68	17.24	8.69
*Capnocytophaga ochracea*	0.00	3.44	0.00	0.00
*Enterobacter amnigenus*	0.00	3.44	0.00	0.00
*Enterobacter kobei*	0.00	0.00	0.00	4.34
*Klebsiella oxytoca*	3.85	0.00	0.00	0.00
*Klebsiella pneumoniae*	0.00	3.44	0.00	4.34
*Burkholderia cepacia*	3.85	0.00	0.00	0.00
*Aeromonas caviae*	0.00	0.00	0.00	4.34
	**34.5**	**31.0**	**17.24**	**21.7**
Gram (−) anaerobes				
*Veillonella parvula*	3.84	3.44	3.47	0.00
*Campylobacter gracilis*	0.00	0.00	0.00	4.35
*Prevotella disiens*	0.00	3.44	0.00	0.00
	**3.84**	**6.89**	**3.47**	**4.35**
Fungi				
*Candida albicans*	11.53	10.34	17.24	21.73

^*∗*^Significance *P* < 0.05 (compared to baseline data from test I).

Test I—sample collected before GC or GA gel application (baseline).

Test II—sample collected 5-6 weeks following GC or GA gel application (final assessment).

**Table 3:** Total amount of isolated microorganisms present in swabs from oral cavity of surgical patients using gel without propolis (GC preparation) and gel with 3% EEP-B (GA preparation).

	Gram (+) cocci	Gram (−) cocci	Gram (−) rods	Gram (+) rods and bacilli	Fungi	Total [*n*]
GC preparation						
Test I	12	8	2	1	3	26
Test II	10	7	4	5	3	29

GA preparation						
Test I	13	6	0	5	5	29
Test II	9	2	3	4	5	23

Test I—sample collected before GC or GA gel application (baseline).

Test II—sample collected 5-6 weeks following GC or GA gel application (final assessment).

## References

[1] Mombelli A., Lang N. P. (1992). Antimicrobial treatment of peri-implant infections. *Clinical Oral Implants Research*.

[2] Larrazábal C., García B., Peñarrocha M., Peñarrocha M. (2010). Influence of oral hygiene and smoking on pain and swelling after surgical extraction of impacted mandibular third molars. *Journal of Oral and Maxillofacial Surgery*.

[3] Peñarrocha-Diago M., Sanchis J. M., Sáez U., Gay C., Bagán J. V. (2001). Oral hygiene and postoperative pain after mandibular third molar surgery. *Oral Surgery, Oral Medicine, Oral Pathology, Oral Radiology, and Endodontics*.

[4] Nørholt S. E. (1998). Treatment of acute pain following removal of mandibular third molars: use of the dental pain model in pharmacological research and development of a comparable animal model. *International Journal of Oral and Maxillofacial Surgery*.

[5] Bou-Chacra N. A., Gobi S. S., Ohara M. T., Pinto T. D. J. A. (2005). Antimicrobial activity of four different dental gel formulas on cariogenic bacteria evaluated using the linear regression method. *Revista Brasileira de Ciencias Farmaceuticas*.

[6] Ciancio S. G., Lauciello F., Shibly O., Vitello M., Mather M. (1995). The effect of an antiseptic mouthrinse on implant maintenance: plaque and peri-implant gingival tissues. *Journal of Periodontology*.

[7] Fine D. H., Furgang D., Markowitz K., Sreenivasan P. K., Klimpel K., De Vizio W. (2006). The antimicrobial effect of a triclosan/copolymer dentifrice on oral microorganisms in vivo. *Journal of the American Dental Association*.

[8] Radafshar G., Mahboob F., Kazemnejad E. (2010). A study to assess the plaque inhibitory action of herbal-based toothpaste: a double blind controlled clinical trial. *Journal of Medicinal Plants Research*.

[9] Lee K.-H., Kim B.-S., Keum K.-S. (2011). Essential oil of *Curcuma longa* inhibits *Streptococcus mutans* biofilm formation. *Journal of Food Science*.

[10] Marsh P. D., Percival R. S. (2006). The oral microflora—friend or foe? Can we decide?. *International Dental Journal*.

[11] Aas J. A., Paster B. J., Stokes L. N., Olsen I., Dewhirst F. E. (2005). Defining the normal bacterial flora of the oral cavity. *Journal of Clinical Microbiology*.

[12] Paster B. J., Boches S. K., Galvin J. L. (2001). Bacterial diversity in human subgingival plaque. *Journal of Bacteriology*.

[13] Li X., Kolltveit K. M., Tronstad L., Olsen I. (2000). Systemic diseases caused by oral infection. *Clinical Microbiology Reviews*.

[14] Lockhart P. B., Brennan M. T., Sasser H. C., Fox P. C., Paster B. J., Bahrani-Mougeot F. K. (2008). Bacteremia associated with toothbrushing and dental extraction. *Circulation*.

[15] Bahrani-Mougeot F. K., Paster B. J., Coleman S., Ashar J., Barbuto S., Lockhart P. B. (2008). Diverse and novel oral bacterial species in blood following dental procedures. *Journal of Clinical Microbiology*.

[16] Storoe W., Haug R. H., Lillich T. T. (2001). The changing face of odontogenic infections. *Journal of Oral and Maxillofacial Surgery*.

[17] Daniel Labriola J., Mascaro J., Alpert B. (1983). The microbiologic flora of orofacial abscesses. *Journal of Oral and Maxillofacial Surgery*.

[18] Shenep J. L. (2000). Viridans-group streptococcal infections in immunocompromised hosts. *International Journal of Antimicrobial Agents*.

[19] Chuang S.-K., Perrott D. H., Susarla S. M., Dodson T. B. (2008). Risk Factors for Inflammatory Complications Following Third Molar Surgery in Adults. *Journal of Oral and Maxillofacial Surgery*.

[20] Roda R. P., Jiménez Y., Carbonell E., Gavaldá C., Muñoz M. M., Pérez G. S. (2008). Bacteremia originating in the oral cavity. A review. *Medicina Oral, Patologia Oral y Cirugia Bucal*.

[21] American Association of Oral and Maxillofacial Surgeons The Oral and Maxillofacial Surgeon. http://www.aaoms.org/.

[22] Viuda-Martos M., Ruiz-Navajas Y., Fernández-López J., Pérez-Álvarez J. A. (2008). Functional properties of honey, propolis, and royal jelly. *Journal of Food Science*.

[23] Marcucci M. C. (1995). Propolis: chemical composition, biological properties and therapeutic activity. *Apidologie*.

[24] Kumazawa S., Hamasaka T., Nakayama T. (2004). Antioxidant activity of propolis of various geographic origins. *Food Chemistry*.

[25] Seidel V., Peyfoon E., Watson D. G., Fearnley J. (2008). Comparative study of the antibacterial activity of propolis from different geographical and climatic zones. *Phytotherapy Research*.

[26] Bankova V., Christov R., Kujumgiev A., Marcucci M. C., Popov S. (1995). Chemical composition and antibacterial activity of Brazilian propolis. *Zeitschrift für Naturforschung C*.

[27] Silici S., Kutluca S. (2005). Chemical composition and antibacterial activity of propolis collected by three different races of honeybees in the same region. *Journal of Ethnopharmacology*.

[28] Tanasiewicz M., Skucha-Nowak M., Dawiec M., Król W., Skaba D., Twardawa H. (2012). Influence of hygienic preparations with a 3% content of ethanol extract of brazilian propolis on the state of the oral cavity. *Advances in Clinical and Experimental Medicine*.

[29] Dziedzic A., Kubina R., Wojtyczka R. D., Kabała-Dzik A., Tanasiewicz M., Morawiec T. (2013). The antibacterial effect of ethanol extract of polish propolis on mutans streptococci and lactobacilli isolated from saliva. *Evidence-Based Complementary and Alternative Medicine*.

[30] Park Y. K., Alencar S. M., Aguiar C. L. (2002). Botanical origin and chemical composition of Brazilian propolis. *Journal of Agricultural and Food Chemistry*.

[31] Santos V. R., Sakagami H. (2012). Propolis: alternative medicine for the treatment of oral microbial diseases. *Alternative Medicine*.

[32] Park Y. K., Paredes-Guzman J. F., Aguiar C. L., Alencar S. M., Fujiwara F. Y. (2004). Chemical constituents in *Baccharis dracunculifolia* as the main botanical origin of southeastern Brazilian propolis. *Journal of Agricultural and Food Chemistry*.

[33] Szliszka E., Kucharska A. Z., Sokół-Łętowska A., Mertas A., Czuba Z. P., Król W. (2013). Chemical composition and anti-inflammatory effect of ethanolic extract of Brazilian green propolis on activated J774A.1 macrophages. *Evidence-Based Complementary and Alternative Medicine*.

[34] Skaba D., Morawiec T., Tanasiewicz M. (2013). Influence of the toothpaste with Brazilian ethanol extract propolis on the oral cavity health. *Evidence-Based Complementary and Alternative Medicine*.

[35] Magro-Filho O., de Carvalho A. C. (1994). Topical effect of propolis in the repair of sulcoplasties by the modified Kazanjian technique. Cytological and clinical evaluation. *The Journal of Nihon University School of Dentistry*.

[36] Santos F. A., Bastos E. M. A., Uzeda M. (2002). Antibacterial activity of Brazilian propolis and fractions against oral anaerobic bacteria. *Journal of Ethnopharmacology*.

[37] Feres M., Figueiredo L. C., Barreto I. M. Q., Coelho M. H. M., Araujo M. W. B., Cortelli S. C. (2005). In vitro antimicrobial activity of plant extracts and propolis in saliva samples of healthy and periodontally-involved subjects. *Journal of the International Academy of Periodontology*.

[38] Bruschi M. L., Jones D. S., Panzeri H., Gremião M. P. D., De Freitas O., Lara E. H. G. (2007). Semisolid systems containing propolis for the treatment of periodontal disease: in vitro release kinetics, syringeability, rheological, textural, and mucoadhesive properties. *Journal of Pharmaceutical Sciences*.

[39] Al-Sultan F. A., Mustafa L. A., Al-Niaimi A. I. (2006). Aqueous extracts of propolis and miswak as topical medicament to improve post-operative outcome after surgical removal of impacted lower third molar. *Al-Rafidain Dental Journal*.

[40] Anauate-Netto C., Marcucci M. C., Paulino N. (2013). Effects of typified propolis on mutans streptococci and lactobacilli: a randomized clinical trial. *Brazilian Dental Science*.

[41] Anauate-Netto C., Anido-Anido A., Lewgoy H. R. (2014). Randomized, double-blind, placebo-controlled clinical trial on the effects of propolis and chlorhexidine mouthrinses on gingivitis. *Brazilian Dental Science*.

[42] Instituto Nacional da Propriedade Industrial in Brazil Formulations with propolis for dental use. http://www.inpi.gov.br.

[43] Menezes H., Bacci M., Oliveira S. D., Pagnocca F. C. (1997). Antibacterial properties of propolis and products containing propolis from Brazil. *Apidologie*.

[44] Rezende G. P., Pimenta F. C., Costa L. R. (2006). Antimicrobial activity of two Brazilian commercial propolis extracts. *Brazilian Journal of Oral Sciences*.

[45] Koç A. N., Silici S., Kasap F., Hörmet-Öz H. T., Mavus-Buldu H., Ercal B. D. (2011). Antifungal activity of the honeybee products against *Candida* spp. and *Trichosporon* spp.. *Journal of Medicinal Food*.

[46] Lotfy M. (2006). Biological activity of bee propolis in health and disease. *Asian Pacific Journal of Cancer Prevention*.

[47] Król W., Scheller S., Czuba Z. (1996). Inhibition of neutrophils' chemiluminescence by ethanol extract of propolis (EEP) and its phenolic components. *Journal of Ethnopharmacology*.

[48] Krol W., Czuba Z., Scheller S., Gabrys J., Grabiec S., Shani J. (1990). Anti-oxidant property of ethanolic extract of propolis (EEP) as evaluated by inhibiting the chemiluminescence oxidation of luminol. *Biochemistry International*.

[49] Araujo M. A. R., Libério S. A., Guerra R. N. M., Ribeiro M. N. S., Nascimento F. R. F. (2011). Mechanisms of action underlying the anti-inflammatory and immunomodulatory effects of propolis: a brief review. *Brazilian Journal of Pharmacognosy*.

[50] Wang L., Tu Y.-C., Lian T.-W., Hung J.-T., Yen J.-H., Wu M.-J. (2006). Distinctive antioxidant and antiinflammatory effects of flavonols. *Journal of Agricultural and Food Chemistry*.

[51] Tan-No K., Nakajima T., Shoji T. (2006). Anti-inflammatory effect of propolis through inhibition of nitric oxide production on carrageenin-induced mouse paw edema. *Biological and Pharmaceutical Bulletin*.

[52] Woo K. J., Jeong Y.-J., Inoue H., Park J.-W., Kwon T. K. (2005). Chrysin suppresses lipopolysaccharide-induced cyclooxygenase-2 expression through the inhibition of nuclear factor for IL-6 (NF-IL6) DNA-binding activity. *FEBS Letters*.

[53] Lopes-Rocha R., de Miranda J. L., Lima N. L., Ferreira F. O., Marinho S. A., Verli F. D. (2012). Effect of topical propolis and dexamethasone on the healing of oral surgical wounds. *Wound Healing Southern Africa*.

[54] Sánchez R., Mirada E., Arias J., Paño J. R., Burgueño M. (2011). Severe odontogenic infections: epidemiological, microbiological and therapeutic factors. *Medicina Oral, Patologia Oral y Cirugia Bucal*.

[55] Lewis M. A. O., MacFarlane T. W., McGowan D. A. (1990). A microbiological and clinical review of the acute dentoalveolar abscess. *British Journal of Oral and Maxillofacial Surgery*.

[56] Rega A. J., Aziz S. R., Ziccardi V. B. (2006). Microbiology and antibiotic sensitivities of head and neck space infections of odontogenic origin. *Journal of Oral and Maxillofacial Surgery*.

[57] Sixou J.-L., Magaud C., Jolivet-Gougeon A., Cormier M., Bonnaure-Mallet M. (2003). Microbiology of mandibular third molar pericoronitis: Incidence of *β*-lactamase-producing bacteria. *Oral Surgery, Oral Medicine, Oral Pathology, Oral Radiology, and Endodontics*.

[58] Stefanopoulos P. K., Kolokotronis A. E. (2004). The clinical significance of anaerobic bacteria in acute orofacial odontogenic infections. *Oral Surgery, Oral Medicine, Oral Pathology, Oral Radiology and Endodontology*.

[59] Morawiec T., Dziedzic A., Niedzielska I. (2013). The biological activity of propolis-containing toothpaste on oral health environment in patients who underwent implant-supported prosthodontic rehabilitation. *Evidence-Based Complementary and Alternative Medicine*.

[60] Malhotra N., Rao S. P., Acharya S., Vasudev B. (2011). Comparative in vitro evaluation of efficacy of mouthrinses against *Streptococcus mutans*, *Lactobacilli* and *Candida albicans*. *Oral Health & Preventive Dentistry*.

[61] Koru O., Toksoy F., Acikel C. H. (2007). In vitro antimicrobial activity of propolis samples from different geographical origins against certain oral pathogens. *Anaerobe*.

[62] Hayashi K., Komura S., Isaji N., Ohishi N., Yagi K. (1999). Isolation of antioxidative compounds from Brazilian propolis: 3,4-dihydroxy-5-prenylcinnamic acid, a novel potent antioxidant. *Chemical and Pharmaceutical Bulletin*.

[63] Simões L. M. C., Gregório L. E., Da Silva Filho A. A. (2004). Effect of Brazilian green propolis on the production of reactive oxygen species by stimulated neutrophils. *Journal of Ethnopharmacology*.

